# Mass Spectrometry-Based Proteomics in Molecular Diagnostics: Discovery of Cancer Biomarkers Using Tissue Culture

**DOI:** 10.1155/2013/783131

**Published:** 2013-03-17

**Authors:** Debasish Paul, Avinash Kumar, Akshada Gajbhiye, Manas K. Santra, Rapole Srikanth

**Affiliations:** National Centre for Cell Science, University of Pune Campus, Ganeshkhind, Pune, Maharashtra 411007, India

## Abstract

Accurate diagnosis and proper monitoring of cancer patients remain a key obstacle for successful cancer treatment and prevention. Therein comes the need for biomarker discovery, which is crucial to the current oncological and other clinical practices having the potential to impact the diagnosis and prognosis. In fact, most of the biomarkers have been discovered utilizing the proteomics-based approaches. Although high-throughput mass spectrometry-based proteomic approaches like SILAC, 2D-DIGE, and iTRAQ are filling up the pitfalls of the conventional techniques, still serum proteomics importunately poses hurdle in overcoming a wide range of protein concentrations, and also the availability of patient tissue samples is a limitation for the biomarker discovery. Thus, researchers have looked for alternatives, and profiling of candidate biomarkers through tissue culture of tumor cell lines comes up as a promising option. It is a rich source of tumor cell-derived proteins, thereby, representing a wide array of potential biomarkers. Interestingly, most of the clinical biomarkers in use today (CA 125, CA 15.3, CA 19.9, and PSA) were discovered through tissue culture-based system and tissue extracts. This paper tries to emphasize the tissue culture-based discovery of candidate biomarkers through various mass spectrometry-based proteomic approaches.

## 1. Introduction

Cancer is a genetically and clinically diverse disease. The concept of early detection has attracted the attention of both physicians and researchers for decades and thus evolved the concept of “Biomarker” [1]. According to the definition of National Cancer Institute (USA), “biomarker is a biological molecule found in blood, other body fluids, or tissues that is a sign of a normal or abnormal process, or of a condition or disease.” The ideal biomarker should be easily detectable, highly sensitive and specific for its target phenotype as well as economically feasible [2]. A biomarker may be used to monitor the body responses to a treatment for a disease or condition. It is also referred to as a molecular marker or biosignature. It can be any molecule like DNA, RNA, proteins, or metabolites [3]. Although the survival rate of cancer patients has increased in the last 20 years, newer diagnostic methods with improved sensitivity and specificity are essential for the proper detection and prognosis of this fatal disease.

**Figure 1:**
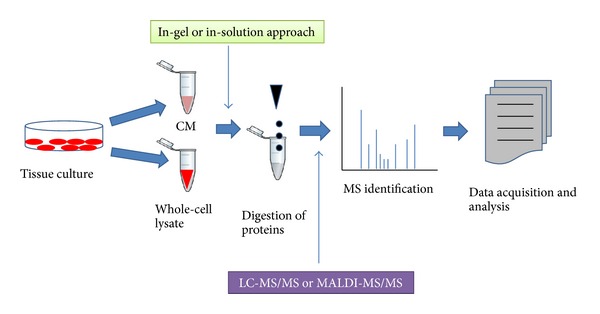
An overview of biomarker discovery using tissue culture. Cancer cells are cultured in plates. The CM as well as cells is collected separately. Extracted proteins from each fraction are processed for either in-gel or in-solution digestion followed by the detection of peptides by mass spectrometric approach. Data analysis leads to detection of candidate biomarkers.

Discovery of biomarkers through the analysis of patient serum or tissue is a conventional approach being used since the beginning of diagnosis of cancer, but the broad range of serum proteome and availability of patient tissue samples are the major hurdles. Thus, the use of tumor cell lines becomes an attractive option for the study and discovery of candidate biomarkers since the cells possess a rich source of secreted as well as cellular proteins. Secretome comprising the secretory proteins in the culture media, also referred to as conditioned media (CM), serve as a potent source for biomarkers due to ease and effectiveness of detection; however, nowadays even cellular proteins are also providing important information about disease conditions. Thus, this model system can serve as an early provider of potential biomarkers. An overview of tissue culture-based model system for candidate cancer biomarker discovery is represented in Figure 1. A number of studies have used the cell culture-based system to identify the potential biomarkers [4–6]. The clinical significance of using cell lines to understand biological functions lies in the fact that they can be examined through various techniques and that they display the same heterogeneity as the primary tumors as well as different grades [7, 8].

 We have witnessed a tremendous improvement in the past decade in the field of high-throughput research that heralds the initiation of a new era in the area of biological science research. Almost all proteomic biomarker discovery platforms use mass spectrometry (MS) as the central technique in association with other proteomic approaches. MS has certain advantages like prediction of molecular mass with the highest specificity and sensitivity with the use of smallest amount of sample [9–11]. Different mass spectrometry-based proteomic approaches have been used to identify biomarkers from various sources and are broadly classified into two categories: gel-based (2-DE and 2D-DIGE) and gel-free (SILAC, iTRAQ) techniques [12–14]. Detection of biomarkers through two-dimensional gel electrophoresis (2-DE) is the most widely used gel-based approach [15]. Improvements over the years have provided us with a more sensitive and high-throughput gel-based technique termed as two-dimensional difference gel electrophoresis (2D-DIGE). This is based on the differential excitation-emission properties of fluorescent dyes such as Cy2, Cy3, and Cy5 [16]. Apart from the gel-based techniques, gel-free techniques have been dominating the field of biomarker discovery in the last decade. Stable isotope labelling by amino acids in cell culture (SILAC), which relies on the incorporation of amino acids with substituted stable isotopic nuclei such as H^2^, C^13^, and N^15^, is highly suitable for tissue culture-based model system [17]. Another very sensitive gel free technique known as isobaric tags for relative and absolute quantitation (iTRAQ) is also a method of choice [18]. 

Moreover, these MS-based proteomic tools have advanced satisfyingly since the last decade and hence have become capable of simultaneously identifying thousands of proteins even from very small amounts. MS advancement has helped enormously in the identification and delivery of candidate biomarkers for cancer diagnosis, prognosis and monitoring of treatment regimen. 

## 2. Mass Spectrometry-Based Proteomics 

**Figure 2:**
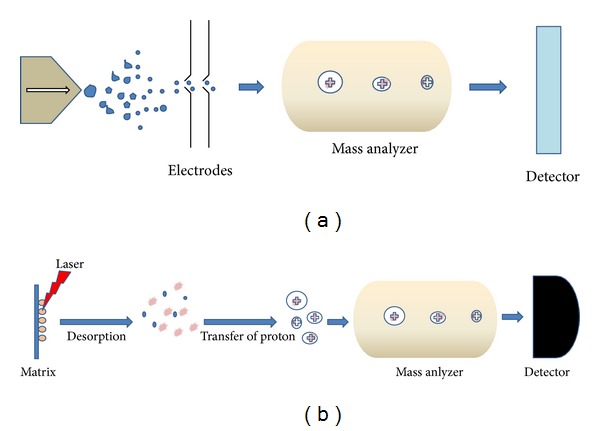
(a) A schematic representation of ESI-MS—solvent along with sample flows from the needle with electrical potential generating charged droplets. The droplets carry the sample, and they are desolvated by applying heat and nebuliser gas to produce ions. These ions are now separated according to *m/z* ratio in the mass analyzer and registered by detector. (b) A schematic representation of MALDI-MS—the sample is mixed with the matrix and allowed to crystalize on the MALDI plate, when the laser hits the sample-matrix mixture on the plate, matrix absorbs the energy of the laser to get vaporized along with samples. Next, the charge exchange takes place from matrix and sample ions are generated.

MS has increasingly become the method of choice for all the proteomic approaches available to date. As the name indicates, “mass spectrometry” determines the molecular mass of a charged particle by measuring its mass-to-charge (*m/z*) ratio. Basically, a mass spectrum is a plot of ion abundance versus *m/z.* A mass spectrometer consists of an ion source that converts molecules to ionized analytes, a mass analyser that resolves ions according to *m*/*z ratio*, and a detector that registers the number of ions at respective *m*/*z* value. The mass analyser depends on three key parameters: sensitivity, resolution, and mass accuracy. The sensitivity, resolution, and accuracy of advanced mass spectrometers allow the detection of femtogram levels of individual proteins in complex mixtures. As recognized by the 2002 Nobel Prize in Chemistry, innovation of electrospray ionization (ESI) and matrix-assisted laser desorption/ionization (MALDI) techniques has made it possible to ionize big molecules such as proteins, peptides, and nucleotides for mass spectrometric analysis. ESI generates ions at atmospheric pressure by injecting a solution-based sample through a small capillary (Figure 2(a)). MALDI produces ions by pulsed-laser irradiation of a sample which is cocrystallized with a solid matrix that can absorb the wavelength of light emitted by the laser (Figure 2(b)). Protonation or deprotonation is the main source of charging for the ions generated in ESI/MALDI. MS-based proteomics is a widely used approach to find protein sequence from unknown samples by correlating the sequence ions generated from tandem mass spectral data with sequence information available in protein databases. MS-based proteomics analyses of complex protein mixture usually require a starting amount in the range of 0.1–10 *μ*g, depending on the experimental setup and the type of mass spectrometer used. ESI is playing an increasingly conspicuous role in the study of the protein structure, folding, and noncovalent interactions [19]. Recently, MALDI imaging has allowed biomolecular profiling of tissue sections and single cells [20]. In combination with chromatographic separation techniques, MS is playing an important role in discovering the biomarkers for various diseases. Many research groups have been using MS-based techniques in order to identify potential cancer biomarkers for diagnostic as well as therapeutic purposes [21–25].

## 3. Mass Spectrometry-Based Quantitative Proteomic Strategies towards Biomarker Discovery

 Cancer remains a major cause of mortality worldwide despite the progress in detection, diagnosis, and therapy. Early diagnosis of cancer improves the likelihood of successful treatment and can save many lives. Thus, early diagnostic biomarkers are highly important for detection and diagnosis in cancer, but due to the lower sensitivity and lack of specific biomarkers, there is an urgent need to discover new and better biomarkers that would be helpful in improving cancer diagnosis, prognosis and treatment. Proteomics is the most powerful technique which can help to discover novel candidate biomarkers for cancer. Current progress in proteomics has been largely due to recent advancements in MS-based technologies. This powerful MS-based quantitative proteomic technologies can aid in the identification of all differentially expressed proteins and their posttranslational modifications during cancer progression which can be used as biomarkers for early diagnosis and monitoring disease treatment in cancers. Moreover, the candidate biomarkers for other diseases, like diabetes, cardiovascular, and so forth, are also discovered with the help of these techniques [26, 27]. This section focuses on different mass spectrometry-based proteomic strategies and explores their applications in potential biomarker discovery.

## 4. 2D Gel Electrophoresis (2-DE)

**Figure 3:**
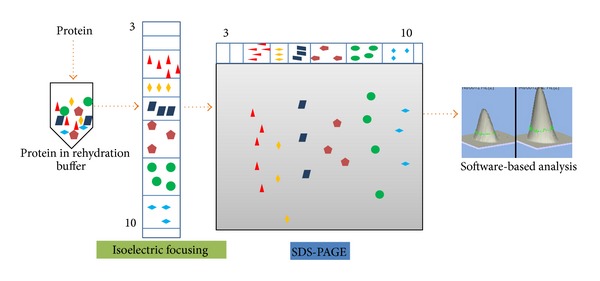
An outline of 2-dimensional gel electrophoresis (2-DE). The extracted proteins are solubilized in rehydration buffer. The proteins are immobilized on IPG strips of different pH ranges depending on the requirement of the experiment. In the first-dimension, the proteins are separated on the basis of their isoelectric points (pI) and are further resolved according to their molecular weight in the second-dimension. Finally, protein spots of interest are excised and subjected to tryptic digestion followed by MS.

The 2-DE method is a primary technique regularly used in proteomic investigations [15]. In this method, extracted proteins are resolved in the first-dimension based on their isoelectric point (pI) followed by molecular weight in the second-dimension (Figure 3). The gels are then stained by either Coomassie Brilliant Blue or silver stain to visualize the protein spots. Using 2-DE software, differentially expressed protein spots are excised and identified by mass spectrometry [28]. This approach could lead to separation and identification of about 2000 unique spots. Using the 2-DE, Braun et al. successfully identified 64 differentially regulated proteins in cancer by mass spectrometry and showed that microfilamental network-associated proteins are frequently downregulated in leukocytes of breast cancer patients [29]. These are functionally important for all central processes and highly relevant for all stages of tumorigenesis-like metastasis [29]. Similarly, Cancemi et al. identified S100 group of proteins that are preferentially expressed in tumor samples than their normal counterpart. They have used breast cancer as subject of study and established for the first time the importance of the S100 group of proteins as potential biomarkers [30]. 

This technique is also being routinely used for the proteomic profiling of cancer cells treated with drugs (*in vitro).* Strong et al. studied the differential regulation of mitochondrial proteome of Adriamycin-resistant MCF-7 breast cancer cells. They have identified 156 unique proteins and established coproporphyrinogen III oxidase and ATP synthase alpha chain to be responsible for the chemotherapeutic resistance [31]. Similar kind of study has been carried out by Lee and coworkers to show hnRNPA2 and GDI2 proteins to be associated with paclitaxel resistance in ovarian cancer cell lines [32]. They have established a paclitaxel resistance subline SKpac from the sensitive counterpart SKOV3 followed by quantitative proteomic analysis and further validated their findings by western blotting. These examples demonstrate the potency of 2-DE approach in the discovery of novel proteins involved in tumorigenesis and chemotherapeutic drug response. However, 2-DE still has its limitations like the inability to resolve too basic, too acidic, and hydrophobic proteins. The ampholytes used for the generation of pH gradient are not stable at extreme acidic and basic pH and are therefore unsuitable for use. In addition, the membrane proteins due to their highly hydrophobic nature pose problems in solubilisation, making them difficult to resolve. Reproducibility and low relative quantification accuracy are other major obstacles which arise due to factors such as run to run variation and limitation of the detection methods available [33]. Requirement of huge amount of sample and inability to detect low abundance proteins is also a major drawback. Though 2-DE has its limitations, still it will be a method of choice for proteomic study because of its robustness and simplicity. 

## 5. 2D Difference Gel Electrophoresis (2D-DIGE)

**Figure 4:**
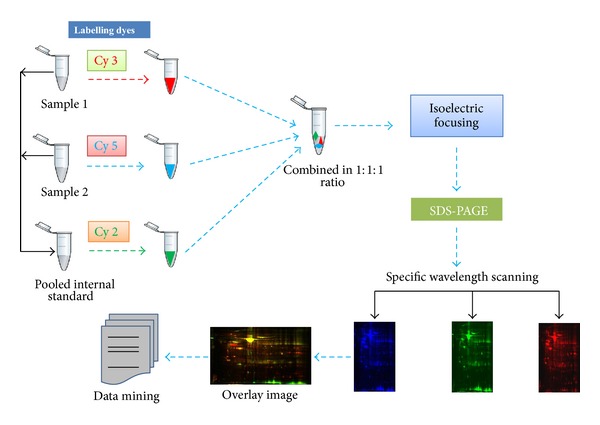
An outline figure of 2D-DIGE. Proteins are extracted from the samples and are labelled with different fluorophores as Cy 3 for sample 1, Cy 5 for sample 2, and Cy 2 for the pooled internal standard. All the samples are resolved in the same 2D gel followed by protein spot pattern detection by scanning the gel in respective wavelength for the Cy dyes; the merging of all of them yields an overlay image consisting of all three Cy dyes. The images are analyzed to get potential candidates of interest.

The 2D-DIGE method is an improved version of 2-DE technique. In this technique, two different protein samples (control and diseased) and one internal control (mixture of control and diseased sample in equal proportion) are labelled with any of the three fluorophores: Cy2, Cy3, or Cy5. These fluorophores have the identical charge and molecular mass but unique fluorescent properties. This allows us to discriminate them during scanning using appropriate optical filters [16, 34]. The labelled samples are then mixed together and separated on a single gel. The best part of this technique is the use of the same internal pool for all the gels that serves as an internal control for normalization (Figure 4) [16, 34]. The gel is scanned by an advanced scanner which can resolve the three different wavelengths: 488 nm (Cy2), 532 nm (Cy3), and 633 nm (Cy5). Each of the samples generates its unique image. This technique eliminates gel-to-gel variation, enhances sensitivity (order of 4 magnitudes), and is less laborious [35, 36]. However, the sample source variation of 2D-DIGE is as vivid as 2-DE. This technique is routinely used for the discovery of candidate biomarkers as well as any quantitative proteomic data generation and therapeutic drug development. Zhang et al. used this technique for the identification of differentially expressed proteins between early submucosal noninvasive and invasive colorectal cancer [37]. They have established a Fischer-344 rat model for the invasive and noninvasive colorectal cancer and found two candidates, transgelin (upregulated) and carbonic anhydrase 2 (CSII) to play significant role in CRC. They have also validated these candidates through fluorescence-based quantitative polymerase chain reaction, western blotting and immunohistochemistry assays [37]. In a similar kind of study, isocitrate dehydrogenase 1 (IDH1) was detected and validated as a potential biomarker for nonsmall cell lung carcinoma [38]. They have identified IDH1 as a potential biomarker in different NSCLC cell lines and further validated it using patient tissue samples via different techniques like western blotting, immunohistochemistry, knockdown assay, and xenograft model. Although the relevance of IDH1 via different genomic and molecular biology techniques is well established now, the basis of its potential was established by this kind of proteomic studies [38]. In another study, Banerjee et al. used 2D-DIGE in combination with MS for the identification of prognostic biomarkers in glioblastoma multiforme using human astrocyte cells and HTB12 human astrocytoma cells [39]. Similarly, Sinclair et al. used this technique to identify the candidate tumor suppressor biomarker in ovarian cancer cell lines: TOV-112D and TOV-21G. They have employed 2D-DIGE and 2D-LC-MS/MS with tandem mass tagging (TMT) to identify potential tumor suppressors in cell lysate [40]. In a separate study, Wilmes et al. compared the proteomic profile of paclitaxel and peloruside-A-treated HL-60 promyelocytic leukemic cells [41]. This technique is a widely used and accepted one in the field of quantitative proteomics. Although the major limitations of 2-DE still apply to 2D-DIGE, but the introduction of more sensitive 2-D DIGE technique has overcome most of the limitations such as requirement of huge amount of sample and inability to detect low abundance proteins. Although in the past decade gel-free techniques have developed immensely, 2D-DIGE has kept its position in proteomic research and will be there for years to come.

## 6. Stable Isotope Labelling by Amino Acids in Cell Culture (SILAC)

**Figure 5:**
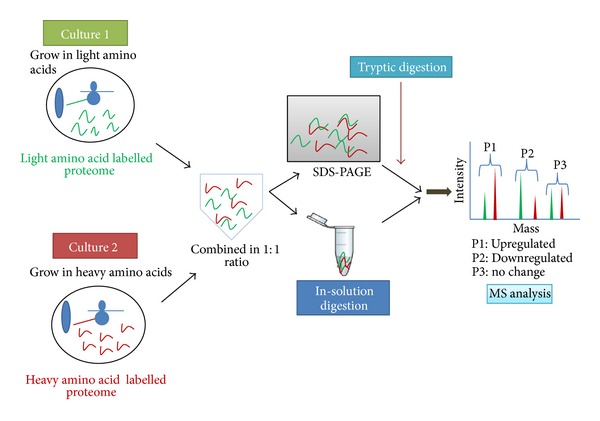
A schematic overview of SILAC. Cells are grown in normal and heavy amino acids containing media for 6 generations to achieve maximal incorporation of heavy amino acids. The proteins are extracted from both populations of cells and mixed in equal proportion and then subjected to either in-gel or in-solution digestion. Relative abundance of the digested peptides is determined from the ratio of heavy-to-light peptide signals as obtained from MS.

The use of quantitative proteomic techniques for the identification of potential biomarkers is a fast gaining ground. For cell culture-based comparative proteomic studies, SILAC is a method of choice [17]. A number of amino acids such as arginine, leucine, and lysine with stable isotope are suitable for use in SILAC, but lysine and arginine are the two most commonly used labelled amino acids. This method solely relies on metabolic incorporation of labelled (heavy) amino acids during cell proliferation. Two different populations of cells (tumor cells and normal cells) are cultured *in vitro* under similar conditions except that tumor cells are grown in media containing heavy isotope of an amino acid (e.g., C^13^ labelled arginine) and the normal cell line is grown in usual media. The cells are allowed to grow as usual for over five to seven passages to ensure >95% labelling [42]. Once the cell lysates are prepared, the samples are combined in a 1 : 1 stoichiometric ratio. Prepared samples are then separated on a SDS-PAGE and further subjected to in-gel trypsin digestion followed by MS analysis. The samples may also be digested in-solution before analysis. During the analysis by mass spectrometer, different isotope composition can be differentiated as the labelled amino acids will induce a shift in the *m/z* ratio in comparison to the unlabelled amino acids. This process ensures that a particular peptide fragment of diseased sample differs from its normal counterpart in *m/z* ratio and hence enabling them to be detected by mass spectrometry (Figure 5). Geiger et al. identified prognostic biomarkers such as IDH2, CRABP2, and SEC14L2 for overall breast cancer survival [43]. They have done the stage-specific analysis of proteome using tissue culture-based model system and further validated them using the patient tissue samples. They validated the candidates via immunohistochemistry and tissue array of human tumor samples. These kinds of holistic studies have helped us to find the potential biomarker for monitoring disease progression and prognosis (CRABP2 and IDH2 are markers of poor prognosis and SEC14L2 is a marker of good prognosis) [43]. Kashyap et al. used SILAC-based proteomic investigation for the discovery of new candidate biomarkers in oral squamous cancer using tissue culture-based system [44]. In a similar type of study, Wang et al. established the regulatory network of karyopherin subunit alpha-2 (KPNA2) as a novel cargo protein in nonsmall cell lung carcinoma (NSCLC) to further establish KPNA2 as a candidate biomarker for NSCLC [45]. In a different kind of approach, Cuomo et al. used this versatile technique for the identification of histone signatures in breast cancer cell lines. They specifically focused on histone H3 and H4 and came up with “breast cancer-specific epigenetic signature,” with implications for the characterization of histone-related biomarkers [46]. Moreover, the use of this technique is no longer confined to *in vitro* cell culture. Recently, the founder of this technique, Matthias Mann, has come up with a variation for the use of SILAC *in vivo* [47]. Here, the authors labelled the mice by continuous feeding of either natural or heavy isotope lysine-containing food for four generations. They isolated blood samples and organs to evaluate the incorporation of heavy isotope and found that all the proteins were labelled in the second generation. Further, they validated their result by comparing the proteomes from platelets, heart and erythrocytes from *β*1-integrin, *β*-parvin, and kindlin-3 deficient mice, respectively [47]. They proposed that it is a novel technique, which can be used to monitor the function(s) of a gene at a proteomic level *in vivo* by generating knockout mouse of that gene. Although *in vivo* SILAC mouse model is a great advancement, the same technique cannot be applied to human subjects. SILAC's advantage lies in the nonrequirement of targeted analysis of specific proteins or peptides, as every peptide is labelled and can be quantified depending on the degree of resolution and instrument sensitivity. It is also more robust and accurate than other quantitative techniques. However, SILAC also has few drawbacks like it cannot be used directly to human tissue samples as well as autotrophic cells (plant cells). Moreover, costly reagents are also an obstacle [48]. Although SILAC has its own set of disadvantages, it has immense potential and is yet to be exploited fully. It is gaining popularity quickly and will continue to be used as a significant tool in quantitative proteomic studies. 

Super-SILAC is an improved version of SILAC. As a single-cell line cannot represent the heterogeneity of tumor tissue, super-SILAC helps to enhance the sensitivity and robustness of tissue culture-based model system for quantitative proteomic approach [49]. This method relies on the use of a mix of several SILAC-labelled cell lines as an internal standard for more comprehensive representation of the tumor proteome. Geiger et al. have used this method to show that it represents the tumor heterogeneity better than SILAC. They have used a panel of breast cancer, glioblastoma, and astrocytoma cell lines that represent the internal standards for these tumor types [50]. Boersema et al. used super-SILAC with LC-MS/MS to identify N-glycosylated proteins in cell secretome and patient blood samples. They have used 11 breast cancer cell lines that represent different stages of breast cancer and took few cell lines representing the super-SILAC mix as internal standard for more accurate quantification. Enriched N-glycosylated proteome mainly comprised the membrane and secretory proteins. They have validated the identified candidates in human blood samples [51]. Lund et al. have used it for the study of metastatic markers in primary tumors. They compared the proteome of tumors derived from inoculation of a panel of isogenic human cancer cell lines with different metastatic capabilities into the mammary fat pad of immunodeficient mice [52]. As of now, it does show a great potential to serve as a relative proteomic quantitation method for understanding molecular aspects of cancer biology and perhaps as a convenient approach for candidate biomarker discovery. Due to its high accuracy and low error rate, it is becoming the method of choice in quantitative proteomics. 

## 7. Isobaric Tagging Reagent for Absolute Quantitation (iTRAQ)

**Figure 6:**
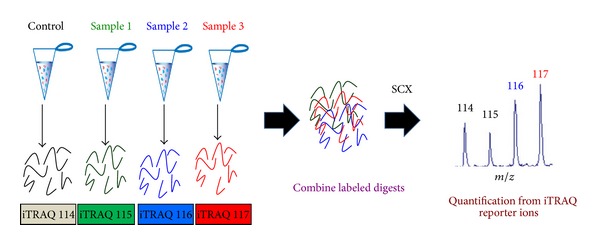
Experimental outline of iTRAQ. Proteins are extracted from either tissue samples or cultured cells and subjected to proteolytic digestion. The digested peptides are then labelled with isobaric tags followed by the pooling of the samples. The samples are then fractioned through SCX followed by tandem mass spectrometry analysis.

Another popular and comprehensive quantitative technology is Isobaric Tagging Reagent for Absolute Quantitation (iTRAQ) introduced by Ross et al. [18]. iTRAQ label consists of a reporter group (variable mass of 114–117 Da), a balance group, and an amine-reactive group that reacts at lysine side chains and NH_2_-terminal. In iTRAQ, samples are labelled after trypsin digestion with four independent iTRAQ reagents. The labelled samples are pooled and the tagged peptides are fractionated by strong cation exchange (SCX) chromatography, and each desalted fraction is subjected to tandem mass spectrometry [53]. The reporter groups of the iTRAQ reagent generate reporter ions for each sample with mass/charge (*m/z*) of 114, 115, 116, and 117 during MS/MS. These reporter ions allow the differentiation of the different samples in MS and furnish the necessary quantitative information (Figure 6). Recently, electrostatic repulsion-hydrophilic interaction chromatography (ERLIC) and off-gel fractionation have evolved as an alternative to the cumbersome process of SCX chromatography [54, 55]. ERLIC method separates peptides on the basis of electrostatic repulsion and hydrophilic interaction and is found to be increasing the proteome coverage [54]. In off-gel fractionation, the samples are rehydrated on a gel strip and further separated up to 24 fractions according to pI [55]. iTRAQ method can also be improved to perform absolute quantification by adding internal standard peptide. Recently, Eight-plex iTRAQ reagents have also become commercially available that allows the quantification of eight different samples in a single run. The advantage of iTRAQ labelling is that signal obtained from combined peptides enhances the sensitivity of detection in MS and MS/MS. However, the variability in labelling efficiencies and the costly reagents are major limitations of this high fidelity technique [56]. The use of this powerful technique is gradually becoming the method of choice in the field of biomarker discovery. In a study by Rehman and coworkers, this technique was used for candidate biomarker discovery associated with metastasis using both patient sample as well as prostate cancer cell lines [57]. They have pooled serum samples from three different stages of prostate cancer patient group as nonprogressing samples, progressing samples, and metastatic samples followed by identification of a set of potential prognostic biomarkers that may be involved in disease progression and metastasis. They have identified eEF1A1 as a novel candidate biomarker significantly showing increase in all three groups of samples when compared to benign prostatic hyperplasia (BPH) samples. They have also validated their results in 11 frequently used prostate cancer cell lines to show eEF1A1 expression validation both at the translational and transcriptional levels. Further, they have also identified C-reactive protein (CRP) that is already established as a potential marker for bone metastatic prostate cancer [57]. In a similar type of study, metastasis-related candidate biomarkers have been identified in colorectal cancer cell lines. They labelled the whole-cell lysates of SW480 (primary cell line) and SW620 (lymph node metastatic variant of SW480) with 4-plex iTRAQ followed by 2D-LC and MALDI-TOF/TOF to identify *β*-catenin and calcyclin binding protein (CacyBP) as differentially expressed. CacyBP degrades *β*-catenin. Thus, these two proteins show a very nice inverse correlation in the progression of metastasis and hence are potential candidate biomarkers [58]. In another study, it has been used for the profiling of tyrosine phosphorylation level in breast cancer progression using MCF10AT breast cancer cell line [59]. Using complementary MALDI- and ESI-based mass spectrometry, they have identified 57 unique proteins comprising tyrosine kinases, phosphatases, and other signaling network proteins that might play significant role during disease progression. For the first time, they have identified SLC4A7 (sodium bicarbonate cotransporter) and TOLLIP (Toll interacting protein) as novel tyrosine kinase substrates associated with cancer development providing valuable insights into the disease progression [59].

## 8. Label-Free Techniques

To overcome the difficulties in labelling techniques such as high cost of the reagents, higher concentration of sample requirement, and incomplete labelling, researchers are turning to mass spectrometry-based label-free shotgun proteomic technology. It is a very high throughput technique that opens up a new era in the discovery of potential biomarkers. Label-free technology is based on the assumption that the peak area of a peptide in the chromatogram is directly proportional to its concentration [60, 61]. This strategy is generally based on two classes of measurements; the first is based on the measurements of ion intensity changes like peptide peak areas or peak heights in chromatogram, and the second is the spectral counting in the MS/MS analysis. Recently, label-free approaches have been used for absolute quantification in addition to the relative quantification of peptides/proteins. Initially, protein abundance was estimated using protein abundance index (PAI), but later on it was converted to exponentially modified PAI (emPAI) which is routinely used for determining absolute protein abundance. Recently, a modified way of spectral counting termed absolute protein expression (APEX) profiling has been used to measure the absolute protein concentration. Decyder MS from GE Healthcare, Protein Lynx from Waters, and SIEVE from Thermo Electron are some of the commercially available softwares for label-free analysis. This technology is applied for candidate biomarker discovery mostly using clinical samples. Ishihara et al. have used it to identify N-glycoproteins as potential biomarkers in hepatocellular carcinoma [62]. Similarly, it was used by Old and coworkers to identify differentially expressed proteins in K562 human erythroleukemia cells [63]. They used peptide spectral counts and LC-MS/MS to study the simulation effect under different conditions that promote cell differentiation by mitogen-activated protein kinase pathway activation [63]. 

 Like other techniques, this technique also has its own advantages and limitations. It seems to be a promising technique for shotgun quantitation and cheap, simplistic, and less complicated in terms of analysis. The limitation of this technique is redundancy in detection which may arise from the peptides which are shared between more than one protein, leading to the suppression effect. In addition, label-free quantification methods suffer from less accurate, semiquantitative, and are not suitable for low abundance and short proteins. Another drawback is in the normalization of the data while exploring multiple samples in multiple reactions [23, 60, 61]. While considering this technique for the quantitation, one should consider that the correlation of MS/MS spectra with a protein is only an approximation owing to the errors arising due to false identification. Proteins of low abundance could still be present in the sample in spite of the spectral count being zero and also larger proteins can give rise to more tryptic digests, hence more spectral counts. The fact that the signal for a given peptide is governed by many factors like efficiency of fragmentation and ionization in electrospray should also be taken into consideration. Thus, the spectra in MS/MS accounting for the identification of a protein can only be used as an indication of its abundance in the sample [64]. These limitations have left us with the scope for more improvement. 

## 9. Stable Isotope Dilution Mass Spectrometry (SID-MS)

In contrast to the relative quantification proteomic approaches, tandem mass spectrometry-based selected reaction monitoring (SRM), and multiple-reaction monitoring (MRM) techniques have been used for absolute quantification of proteins in combination with stable isotope dilution. This MS-based absolute quantification method relies on the incorporation of known quantities of isotope-labelled standards, which display very similar chromatographic properties to the target compounds but can be distinguished by their difference in *m/z* [65]. This isotope dilution method is generally a targeted approach which is focused on a limited set of proteins. In this method, first initial analysis requires identification of signature peptides for targeted proteins followed by an internal standardization performed by spiking stable isotope-labelled peptides into the samples in defined amount before analysis. Quantification is performed by comparing the peak height or peak area in the extracted ion chromatograph of the isotope-labelled and the native forms of a signature peptide [65]. The major advantage of this method is good linearity and excellent precision, but the accuracy and ability to determine the true abundance of target protein strongly depend on the choice of signature peptide selected and the purity of internal standard. The disadvantage of this method is that it is limited to small number of proteins because suitable internal standards need to be purchased/synthesized. The second disadvantage is that this kind of experiments can be preferably done in triple quadrupole mass spectrometers but not all available tandem mass spectrometers. First time, Kippen and coworkers used this method for precise determination of insulin, C-peptide, and proinsulin levels in blood of nondiabetic and type II individuals [66]. Gerber et al. successfully used this method for absolute quantification of proteins and phosphoproteins from cell lysates [67]. Kuzyk and coworkers used this technique to develop a method for the quantitation of 45 serum proteins in human plasma [68]. Recently, Jiang et al. quantified endogenous cystic fibrosis transmembrane conductance regulator (CFTR) in HT29 and BHK cells using MRM-MS and oxygen stable isotope dilution [69]. Apart from these notable studies, SID-MS has been used for the quantification and verification of potential biomarkers in pancreatic [70], prostate cancer [71], and cardiovascular diseases [72]. SID-MS-based quantification is filling the gap between the discovery and validation phases, which may promote potential biomarkers towards clinical trials and thereby their development as diagnostic tools. 

## 10. Role of Tissue Culture and Proteomics in Candidate Biomarker Discovery

Despite the development of the omic technologies, the search for candidate biomarkers that would provide detailed information on diagnosis, prognosis, and disease monitoring has remained largely elusive. The serum proteome of patient samples is largely (>95%) covered by the most abundant 20 proteins and the potential biomarkers are from the remaining 5% of the proteins, thereby yielding very few cancer biomarkers which are in current clinical use [73]. The availability and number of patient tissue samples are also a limitation. Therefore, researchers thought of an alternative approach comprising tissue culture-based candidate biomarker discovery systems to gain insight into different cancers.

The cancer proteome can be classified into two broad groups: secretome and cellular proteome. Secretome, comprising the secretory proteins, plays important roles in vital cellular functions and they can act locally as well as systemically. The secretome reflects the functionality of a cell in a given environment [74]. The proteins or their fragments are secreted from cancer cells into the media termed as conditioned media (CM). Therefore, secretory proteins can function as novel candidate tumor markers for different cancers and can be extracted from tissue culture media of human cancer cell lines. CM, as a source for potential biomarkers, is increasingly becoming popular as revealed by the surge in the number of recent publications [75–77]. On the other hand, analysis of cellular proteome has also given insight into the pathogenesis and has helped us to come up with candidate biomarkers such as Hv1 (voltage-gated proton channel) [78]. The authors reported that Hv1 is specifically expressed in highly metastatic human breast tumor tissues and cell lines and that its level significantly correlated with tumor size, classification, and disease-free survival [78, 79].

Breast cancer cell lines, specifically MCF7, have been widely used as a model to explore potential breast cancer biomarkers [80]. Jung et al. identified potential biomarkers in lung cancer using tissue culture-based approaches [81]. It is becoming increasingly clearer that cell lines are as heterogeneous as primary tumors [8].

Although the *in vitro* cell culture model provides us with great advantages, it also has its own set of disadvantages as reported by Kulasingam and Diamandis, like a single-cell line has multiple variants that makes this system complex. Moreover, it is yet to reach the stage where it can mimic the tumor microenvironment as well as its real characteristics, and others [6]. The three-dimensional (3D) cell culture techniques have made the things more reliable and versatile because of mimicking the *in vivo* conditions [82]. Therefore, their usage for candidate biomarker discovery is more relevant. Moreover, the field of drug discovery and disease prevention largely depends on the tissue culture-based model system, majorly relying on high-throughput proteomics techniques, because there is no scope for direct human trials of newly developed lead molecules [83, 84]. Recently, there have been a lot of reports where researchers have tried to find out the working mechanism of a drug through proteomic, genomic, and many other techniques [85, 86]. Currently, there is a major concern regarding the drug resistance, which implies the nonresponsiveness of a disease for a certain drug at its working concentration. Researchers are relying heavily on robust proteomic approaches for finding the probable “culprits” for this drug resistance [87–91]. The studies using tissue culture-based candidate biomarker discovery platform are shown in Table 1. 

## 11. Advantages of Tissue Culture in Biomarker Discovery and Diagnosis

Cancer cell lines are the most widely used models to study the deregulation in cancer as well as the identification of potential biomarkers for the early detection and prognosis of cancer [92, 100]. Both the cellular milieu and conditioned medium (CM) serve as a rich source of potential biomarkers. The clinical relevance of using cell lines is already well established [101, 102]. As PSA (prostate-specific antigen), the existing biomarker for detection of prostate cancer poses problems; there is a need for a more accurate biomarker. Qian et al. identified Spondin 2 (Spon-2) as a candidate biomarker for prostate cancer [92]. They first identified the extra-cellular proteins by 2-DE coupled with LC-MS/MS. Further, they concentrated on Spon-2 as it was consistently overexpressing in prostate cancer cell secretome, and then they validated their findings in human prostate cancer tissue samples. Moreover, they have checked the sensitivity and specificity of Spon-2 by receiver operator characteristic (ROC) curve analysis. Spon-2 also out rated PSA in the patient samples in terms of sensitivity and specificity [92]. Similarly, Lee et al. showed high-mobility group protein B1 (HMGB1) as a better prognostic marker over carcinoembryonic antigen (CEA) for colorectal carcinoma [102]. They have used 10 colon cancer cell lines along with a normal colon cell line CCD18Co and detected the presence of HMGB1 in the secreted medium. Further, they validated their findings in patient sera also. They have proven the diagnostic value of HMGB1 in a cohort of 219 colorectal patient samples along with 75 control samples. HMGB1 showed more stage-specific diagnosis value than CEA. When HMGB1 and CEA are combined, the overall diagnostic sensitivity is improved when compared to CEA alone (42% versus 25.6%) and the stage 1 cancer diagnosis (47% versus 5.9%) [102]. This kind of study sets the platform for the identification of potential new prognostic biomarkers that might be a tedious job using patient samples directly. The cell culture-based model system possesses its own uniqueness and benefits. The availability and the number of patient tissue samples always present a challenge for the researchers at least in countries with poor public awareness. This is where the easy availability of cell lines (cancerous and normal) comes in. They can also be easily propagated compared to the patient sample. The other advantage is that the cell culture-based model system is cost effective compared to the patient sample system. This system also has the versatility that patient sample system does not have. The cell culture-based system can be used to check the potential efficacy of a novel lead molecule which can be a prospective drug over various types of cancers. This kind of studies also allows us to get an insight into a drug's mechanism of action. 

CM of cancer cells allows us to search for potential biomarkers at the level of secretome. This approach offers various advantages like removal of the potential infectious sources. Few of the currently available biomarkers also pose problems, as for pancreatic cancer, the best available marker is CA19.9; however, the false positive rates of this marker are high as they are also elevated in nonneoplastic conditions like acute and chronic pancreatitis, hepatitis, and biliary obstruction [103]. The cell secretome possesses a great advantage for the dissection of potential biomarkers; as for the clinical use, the best biomarkers are those that can be detected in body fluids. The cell secretome indirectly represents the proteins that can be found in the body fluids of a patient, so the identified secretory proteins can be a good biomarker. Moreover, the dynamic range of the secretome is very low compared to cell lysate, so it is a better source for the profiling of biomarkers for diseases like cancer. It is a noninvasive method for the detection of biomarkers rather than directly encountering the patient samples, and the availability of so many cell lines that represent the different stages of the disease helps to provide relevant information [104]. It also effectively bypasses the large amount of serum proteins present in the body fluids of patients. Importin alpha subunit-2 (also called KPNA-2) was identified as a candidate biomarker by Wang and colleagues using CL1-0 and CL1-5 lung cancer cell lines. They have integrated the data of cancer cell secretome and transcriptome of adenocarcinoma tissues. Further, they have validated their results by immunohistochemistry, and, moreover, they have shown that KPNA-2 and CEA in combination produce more efficient diagnostic capacity in the patients [105]. A similar approach was taken by Kulasingam and Diamandis to identify the candidate biomarkers in breast cancer cell lines using a panel of three breast cancer cell lines: MCF-10A, BT474, and MDA-MB-468. They have identified low abundant proteins like elafin and kallikrein family of proteins along with highly abundant proteins by using “bottom-up” proteomic technique via 2D-LC-MS/MS on a linear ion trap (LTQ) as a potential drug target as well as candidate biomarker [106]. Using this technique, Ahmed et al. have identified a candidate biomarker, immunoreactive integrin-linked kinase (ILK) for ovarian cancer [107]. Similarly, the cell line established from human prostate cancer was confirmed to release PSA when cultured in serum-free CM [108]. This system can be easily modified to allow us to study the prognostic and diagnostic markers under different conditions. If we wish to study the differential regulation of a candidate prognostic biomarker in different disease conditions, it is only possible by the use of tissue culture model system. Another very important advantage of this system over the patient tissue sample is the relatively easy detection of the less abundant proteins, which are the source of potential biomarkers. In patient sample, the high abundant proteins like albumin and immunoglobulin create problem for the detection of less abundant proteins through high throughput techniques like mass spectrometry. 

Nowadays there are ways to remove high abundant proteins. In most cases, it seems to affect the protein concentration in a big way and people are still trying to find a way to improve this technique. It is often cumbersome to reproduce the data using patient samples because of the heterogeneity. The physical as well as physiological status of the patient plays important role in the tumor biology, but cell culture-based system offers a better way to solve this problem as we have a way to propagate the cells for passages and the results can be more easily reproduced in this system as we can use the same lineage of cells for the study. This system allows us to detect the alterations at proteome level which is also possible for patient sample study but again it is more labour intensive, time consuming, and expensive. In well-defined experimental conditions, the proteome of a cell line should reflect the genetic changes of a cell. To get an *in vivo* insight into the disease, researchers use cancer cell xenograft model system. More recently, 3D cell culture system has become a model of choice. Mikesh et al. have used this system to successfully identify molecular markers associated with melanoma [109]. CD151 was identified as a potential prognostic marker for breast cancer. The researchers have used MDA-MB-231 as a model system. In tumor xenograft model, CD-151 knockdown cells showed reduced tumorigenecity compared to normal tumor cells. CD-151 also affects the tumor vasculature. Moreover, the overall survival rate of CD-151 positive patients was 45.8% compared to CD-151 negative patients. Further, they have deciphered its molecular modulator network to establish it as a novel drug target [110]. In a similar kind of study, Yao et al. have used a lectin affinity-based approach to enrich as well as increase the detectable number of secreted proteins in the CM of cultured tissues followed by LC-MS/MS and identified EFEMP2 as a potential marker for early detection of colorectal carcinoma (CRC). They have also proven it to be superior to CRC biomarker and CEA and validated their results by immunohistochemistry [95]. Lee and coworkers have established H^+^-myo-inositol transporter SLC2A13 as a potential biomarker for cancer stem cell (CSC) in oral squamous cell carcinoma (OSCC) [111]. Head and neck carcinoma is one of the poorly understood cancer and there is a need of biomarkers for its diagnosis and prognosis at early stages. Ralhan et al. have used proteomic-based approaches to identify new potential biomarkers for head and neck carcinoma [96]. They have analyzed the secretory medium of different head and neck cancer cell lines via LC-MS/MS and identified a panel of potential biomarkers. Further, they have validated their results via immunoblotting in patient sera also [96]. Once identified, few of these potential biomarkers can be undertaken for clinical trials to further investigate their potential as biomarkers. Similarly, tissue culture-based model system has been used to mine for potential biomarkers in other cancers as well (Table 1) [93, 94, 97–99].

As stated, there are various advantages of using tissue culture-based candidate biomarker discovery but ultimately the studies have to be carried out in patient sample to validate a potential candidate as a biomarker for diagnosis, prognosis, or disease monitoring. This in no way undermines the potential of tissue culture-based model in potential biomarker discovery as the validation can be achieved by alternative means, but the identification is less cumbersome using this system. The initial studies which include the study of differential expression of a candidate in normal versus malignant cells, their mode of action, or whether they can be used as a potential drug target, have to be done using tissue culture-based model system. It creates the foundation based upon which we can carry forward the hunt for novel biomarkers not only in the field of oncology but also for other prevalent diseases.

## 12. Future Perspective

The inherent capability of mass spectrometry along with its sensitivity, speed, and specificity when combined with tissue culture-based model provides a promising tool for the discovery of candidate potential biomarkers (Table 2). In this paper, we have tried to emphasize the use of tissue culture as model for biomarker discovery along with brief outline of different mass spectrometry-based quantitative proteomic techniques that are routinely used in such studies. With the advancement of mass spectrometry-based proteomic techniques and bioinformatics tools, tissue culture-based model system becomes the most beneficial choice for the identification of potential biomarkers. The CM of these cell lines also serves as a potent source of biomarkers. The contemporary biomarkers generally used in clinics such as carbohydrate antigen CA 125, CA 19.9, and PSA were discovered using cancerous cell lines or tumor extracts [112]. It is likely that the tumor microenvironment or the tumor itself can be a source of biomarkers allowing for better sensitivity and specificity as well as proper diagnosis of the disease. However, in tissue culture-based system, the role of tumor microenvironment in biomarker discovery is yet to reach its peak. The 3D culture methods are currently being used that can be considered as an alternative to 2D culture system which receives criticism for its inability to mimic tumor microenvironment. The CM enriched with secretory proteins is largely used for the identification of potential cancer biomarkers. It acts as a perfect source for the potential biomarkers, and to date the majority of the biomarkers being used clinically are secretory proteins. Proteome profiles of many cancers are influenced by hormones, and tissue culture-based model system serves as a promising approach to study this process. Hormonal stimulation of the cells followed by different gel-based or gel-free proteomic approaches to identify differentially expressed proteins serves as an approach to search for the “cause-effect” candidates. Tissue culture-based model system can also be used in the field of pharmacokinetics and drug discovery. The potential effect of a drug can be assessed by using tissue culture-based system. The differential expression of proteins upon drug treatment also provides the insight into the mechanism of action as well as potential drug targets. Moreover, these differentially expressed proteins can serve as potential biomarkers for drug response in clinics.

There has been a rapid fruitful development of MS-based proteomic techniques in the last decade that has immensely helped researchers in candidate biomarker discovery. First, there was 2-DE and then its 2D-DIGE that enhances the accuracy of quantitation utilizing very littel amount of sample. Now, there are techniques like SILAC and iTRAQ which are more advanced versions of labelling techniques in combination with improved chromatographic, and mass spectrometric techniques provide better resolution. Recently, people have started moving towards label-free quantitation, which is the most advanced form of relative quantitation-based proteomic technique. With this advancement, the number of potential biomarkers will certainly increase, but we have to be very careful and critical in choosing the biomarkers that can be used clinically. It is not tough to anticipate more development in the near future that will make tissue culture-based systems for potential biomarker discovery more robust, sensitive, and reliable. This will lead to the discovery of useful biomarkers for patient diagnosis, prognosis, treatment, and monitoring not only for cancer but also for other diseases.

## Figures and Tables

**Table 1:** Tissue culture-based candidate biomarkers discovery in different cancers.

Cancer types	Cell lines used	Biomarker identified	Clinical relevance	Quantitative techniques used	Reference
Breast cancer	21T series of Breast cancer cell lines HMT-3522-S1, MFM223, HCC202 and HCC2218, HCC1599, HCC1143, HCC1937, MCF7, MCF10A, MDA-MB-453	TIMM 17A IDH2, CRABP2, SEC14L2	Disease prognosis Disease progression and monitoring	SILAC and LC-MS/MS SILAC and MALDI-MS/MS	[5] [44]

Prostate cancer	PC3, LnCAP, 22Rv1	Follistatin, chemokine (C-X-C motif) ligand 16, Pentraxin 3, and spondin 2	Disease progression and monitoring	Two-dimensional chromatography and tandem mass spectrometry 2D-DIGE, MALDI-MS/MS	[76] [92]

Lung cancer	1198 and 1170-I, BEAS-2B and 1799 CL1-0 and CL1-5	PGP9.5, TCTP, TIMP-2, and TPI KPNA2	Disease monitoring Disease detection and progression monitoring	2DE and MS SILAC, LC-MS/MS	[81] [46]

Gastric cancer	AGS and MKN7	GRN	Disease detection and monitoring	2D-LC-MS/MS and iTRAQ	[93]

Pancreatic cancer	PANC1, BxPc3, MIA-PaCa2, SU.86.86	Anterior gradient homolog 2, syncollin, olfactomedin-4, polymeric immunoglobulin receptor, and collagen alpha-1(VI) chain	Early disease detection and monitoring	LC-MS/MS, ELISA	[94]

Colorectal cancer	Tumor samples were cultured *in vitro *	EFEMP2	Detection and monitoring	1D-LC-MS/MS	[95]

Head and Neck cancer	SCC4, HSC2, SCC38, and AMOSIII	alpha-enolase, peptidyl prolyl isomerase A/cyclophilin A, 14-3-3 z, heterogeneous ribonucleoprotein K, and 14-3-3 s	Disease detection and progression monitoring	LC-MS/MS, western blot	[96]

Oral cancer	OEC-M1 and SCC4 OEC-M1 and SCC4	Mac-2 BP Guanylate-binding protein 1 (GBP1)	Early detection of disease Disease detection and progression	MALDI-TOF MS 1D and LC-MS/MS	[97] [98]

Renal cell carcinoma	786-O, Caki-1, A498, ACHN OS-RC-2, HK-2, HUVEC	FoxM1	Detection and potential drug target	IHC, western blot, ELISA	[99]

**Table 2:** Different mass spectrometry-based proteomic approaches with its merits, demerits, and compatibility towards tissue culture.

Proteomic approach	Merits	Demerits	Compatibility with tissue culture^a^	References
2-DE	(i) Robust (ii) Simplistic (iii) Highly suitable for MS analysis	(i) Involves large amount of sample (ii) Low throughput (iii) Poor recovery of hydrophobic proteins	∗∗∗	[15, 33]

2D-DIGE	(i) Multiplexed (ii) Better quantitation (iii) Minimized gel to gel variation	(i) Not suitable for MS analysis (ii) Expensive Cy dyes (iii) Poor recovery of hydrophobic proteins	∗∗∗∗	[16, 34]

SILAC	(i) High-throughput (ii) Robust and accurate (iii) Sensitivity and simplicity	(i) Only suitable for tissue culture model (ii) Costly reagents (iii) Not applicable to tissue samples	∗∗∗∗∗	[25, 48]

Super-SILAC	(i) Better representation of tumor heterogeneity (ii) Accurate quantitation (iii) Less error rate	(i) Only suitable to tissue culture model (ii) Costly reagents (iii) Internal standard library required	∗∗∗∗∗	[50]

iTRAQ	(i) Multiplexed (ii) Applicable to versatile samples (iii) Better quantitation	(i) Incomplete labelling (ii) Involves high amount of sample (iii) Expensive reagents	∗∗∗∗	[18, 56]

Label free	(i) Involves less amount of sample (ii) Broader applicability (iii) Avoid labelling	(i) High-throughput instrumentation (ii) Redundancy in detection (iii) Not suitable for low abundant proteins	∗∗∗∗	[61, 64]

SID-MS	(i) Absolute quantitation (ii) Targeted approach (iii) Applicable to versatile samples	(i) Applicable to limited number of proteins (ii) Internal standards are required (iii) Generally used for validation	∗∗∗	[65, 68]

^a^Number of “∗” indicates extent of compatibility.

## Abbreviations

2-DE: 2-dimensional gel electrophoresis
2D-DIGE: 2-dimensional difference gel electrophoresis
APEX: Absolute protein expression
CEA: Carcinoembryonic antigen
CM: Conditioned medium
CRC: Colorectal carcinoma
emPAI: Exponentially modified PAI
ERLIC: Electrostatic repulsion-hydrophilic interaction chromatography
ESI: Electrospray Ionization
iTRAQ: Isobaric tagging reagent for absolute quantitation
LTQ: Linear trap quadrupole
MALDI: Matrix-assisted laser desorption ionization
m/z: Mass to charge
MRM: Multiple-reaction monitoring
PAI: Protein abundance index
PSA: Prostate specific antigen
ROC: Receiver operator characteristic
SDS-PAGE: Sodium dodecyl sulphate-poly acrylamide gel electrophoresis
SID-MS: Stable isotope dilution-mass spectrometry
SILAC: Stable isotope labelling by amino acids in cell culture
SRM: Selected reaction monitoring.
